# Using the egg parasitoid *Anastatus bifasciatus* against the invasive brown marmorated stink bug in Europe: can non-target effects be ruled out?

**DOI:** 10.1007/s10340-018-0969-x

**Published:** 2018-03-21

**Authors:** Judith M. Stahl, Dirk Babendreier, Tim Haye

**Affiliations:** 1 CABI, Rue des Grillons 1, 2800 Delémont, Switzerland; 2 Institute of Ecology and Evolutionary Biology, University of Bremen, Leobener Str. NW2, 28359 Bremen, Germany

**Keywords:** Biological control, Egg parasitoids, Risk assessment, Fundamental host range, *Halyomorpha halys*

## Abstract

The brown marmorated stink bug, *Halyomorpha halys* (Stål) (Hemiptera: Pentatomidae), has been causing massive damage to various fruit and vegetable crops after its arrival in the USA, and more recently in Europe. To provide an alternative control measure to pesticides, the native egg parasitoid *Anastatus bifasciatus* (Geoffroy) (Hymenoptera: Eupelmidae) was considered as a candidate biological control agent for inundative releases in Europe. In the risk assessment study presented here, all nine heteropteran and 14 out of 19 tested lepidopteran non-target species produced viable *A. bifasciatus* offspring. The proportion of *A. bifasciatus* females producing offspring did not differ between non-target and target for 19 out of the 28 non-target species. Larger host eggs corresponded to increased female-biased sex ratio of the offspring as well as an increase in size, particularly for females, with hind tibia lengths varying from 645.5 ± 46 to 1084 ± 28.5 μm. Larger females were also found to have higher offspring production and increased life expectancy. The results of this study confirmed the polyphagous nature of *A. bifasciatus* and suggest that a number of non-target species, including Lepidoptera of conservation interest, may be attacked in the field. Thus, non-target effects cannot entirely be ruled out, but more information is needed from semi-field and field studies to fully assess potential environmental risks due to inundative releases of this native parasitoid.

**Electronic supplementary material:**

The online version of this article (10.1007/s10340-018-0969-x) contains supplementary material, which is available to authorized users.

## Key message

- Potential non-target effects following inundative releases of the European parasitoid *Anastatus bifasciatus* against the invasive *Halyomorpha halys* are unknown.
- No-choice tests with 9 heteropteran and 19 lepidopteran non-target species were conducted in the laboratory.
- Twenty-three non-target species were suitable hosts for *A. bifasciatus*; the proportion of females producing offspring did not differ between non-target and target for 19 out of 28 tested species.
- Non-target effects due to spill-over cannot be ruled out, and further semi-field and field tests are necessary.

## Introduction

The brown marmorated stink bug (BMSB), *Halyomorpha halys* (Stål) (Hemiptera: Pentatomidae), is an Asian species invasive in North America and Europe, and recently established in South America (Faúndez and Rider 2017). Whereas the introduction to North America occurred in the mid-1990s (Hoebeke and Carter 2003), in Europe *H.* *halys* probably arrived in the early 2000s with first individuals found in the area of Zurich, Switzerland (Wermelinger et al. 2008; Haye et al. 2014a, b). Since its arrival, it has established in many other European countries, and bioclimatic models suggest that there is substantial potential for further spread (Kriticos et al. 2017; Zhu et al. 2017). The wide host range of this exotic pest includes economically important crops such as fruit trees, vegetables and leguminous crops (Kobayashi 1967; Lee et al. 2013). In the USA, *H.* *halys* has been causing massive damage to various crops, e.g. apples, peaches, sweet corn and beans (Kuhar et al. 2012; Leskey et al. 2012a, b; Nielsen and Hamilton 2009). In Europe and Eurasia, including northern Italy and western Georgia, severe damage has been observed in pear and hazelnut orchards (Bosco et al. 2017; Maistrello et al. 2017).

Current control strategies mainly rely on the application of broad-spectrum insecticides (Leskey et al. 2012a, b; Nielsen et al. 2008; Kuhar and Kamminga 2017). These, however, often lack efficacy and their increased application led to a resurgence of secondary pests such as woolly apple aphids or European red mite. Populations of those pests were well regulated by natural enemies before the arrival of *H. halys* (Leskey et al. 2012a). This indicates severe pressure on the often well-established IPM systems in European fruit production, which generally rely on selective pesticides that are not harmful to many natural enemies. Hence, the development and implementation of environmentally sound control methods, such as biological control, are urgently needed to provide an alternative solution against *H. halys* in Europe (Haye et al. 2015).

In China, Korea, and Japan, where the pest originates, its populations are primarily regulated by egg parasitoids in the genera *Trissolcus* (Hymenoptera: Scelionidae) and *Anastatus* (Hymenoptera: Eupelmidae) (Arakawa and Namura 2002; Lee et al. 2013; Talamas et al. 2015). In particular, *Trissolcus japonicus* (Ashmead) causes high parasitism in *H. halys* (Yang et al. 2009; Zhang et al. 2017) and thus is being considered as a classical biological control agent. However, if it appears that it is not regarded as specific to the target host and introductions may have adverse effects, augmentative (inoculative or inundative) use of parasitoids native to the invaded area may provide an alternative control strategy (Haye et al. 2015; Roversi et al. 2016).

Studies searching for native egg parasitoids adopting the invasive *H. halys* as new host have been conducted in the USA and Europe exposing viable or freeze-killed sentinel *H. halys* eggs (Abram et al. 2017). In Switzerland and Italy, parasitoids reared from those sentinel *H. halys* eggs exposed on trees mainly in or in the vicinity of fruit orchards and vineyards were *Ooencyrtus telenomicida* (Vassiliev) (frozen eggs) (Roversi et al. 2016), *Trissolcus cultratus* (Mayr) (frozen eggs), and *Anastatus bifasciatus* (Geoffroy) (viable and frozen eggs) (Haye et al. 2015). The latter is the most common parasitoid reared from sentinel *H. halys* eggs in the field. This makes it a candidate for inundative biological control, where large numbers of natural enemies are released for immediate control of a damaging pest population.

The majority of *Anastatus* spp. are primary endoparasitoids of various insect orders (Askew 2005; Jones 1988) such as Hemiptera, Lepidoptera, Blattodea (Narasimham and Sankaran 1982), Orthoptera and Mantodea (Askew 2005). In the past, *Anastatus* sp. (later identified as *A. japonicus* (Hayat 1975)) was released alongside other parasitoids in the USA against the gypsy moth (Crossman 1925), and several *Anastatus* species are currently being tested or used against various pests in different crops around the world. In Australian macadamia orchards, *Anastatus* sp. is used for augmentative biological control of the fruitspotting bug *Amblypelta nitida* Stål and the banana spotting bug *A. lutescens lutescens* Distant (Hemiptera: Coreidae) (Fay and De Faveri 1997; Govender 2015; Huwer et al. 2011). In Nepal, *Anastatus* sp. was released against the citrus green stink bug *Rhynchocoris humeralis* (Thunberg) (Hemiptera: Pentatomidae) (Shrestha 2011). In China, *Anastatus japonicus* Ashmead has been released for several decades to control the lychee stink bug *Tessaratoma papillosa* Drury (Hemiptera: Pentatomidae) (Chen et al. 1990; Huang et al. 1974). Due to its success, *Anastatus* sp. was also mass-released against *H. halys* in the Beijing area, achieving parasitism rates of more than 60% (Hou et al. 2009).

A similar approach could also be possible in Europe, but the knowledge of the biology and ecology of *A. bifasciatus* in Europe is scarce. Genduso (1977) studied *A. bifasciatus* on *Gonocerus acuteangulatus* (Goeze) (Hemiptera: Coreidae) and demonstrated that the parasitoid completed four generations per year on the island of Sicily, Italy. The remaining literature for *A. bifasciatus* consists primarily of host records from across Europe, including more than 30 species in the orders Hemiptera and Lepidoptera (e.g. Herting and Simmonds 1976; Genduso 1977; Jones 1988; compiled by Noyes 2014).

Concerns regarding the safety of classical biological control using invertebrates, particularly the impact on non-target species, have been discussed intensively. As a result, procedures to select the safest biological control agent have been developed that assess the risks they pose beforehand (Bigler et al. 2006; Barratt et al. 2010; Van Lenteren et al. 2006). Lynch et al. (2001) reported that far more inundative releases have led to population-level non-target effects than classical biological control—although some of these effects were only localized and short term. However, their focus was primarily on exotic biological control agents, whereas inundative releases typically involve native natural enemies. Using native natural enemies instead of exotic ones reduces the potential risks of biological control considerably, since the agents are already naturally present. However, continuous inundative releases of native generalist biological control agents can also lead to non-target effects due to overflow into adjacent habitats (van Lenteren and Loomans 2006) and thus, endangered and beneficial species within those habitats could be at risk. Inundative releases of native parasitoids could further facilitate apparent competition between the native and exotic host sharing the same natural enemies (Holt 1977; Holt and Bonsall 2017). Consequently, the potential risks associated with inundative releases should be assessed even for native natural enemies.

The objectives of the present study were to determine the potential risks of inundative releases of *A. bifasciatus* for non-target species and to investigate whether their suitability differs from those of the target *H. halys*.

## Materials and methods

### Rearing of* Halyomorpha halys*

A *H. halys* colony was established from approximately 80 overwintered adults collected in Zurich and Basel, Switzerland, in 2012, and new individuals from both locations were added on an annual basis. The colony was kept in groups of up to 50 *H. halys* in polyester cages (‘BugDorm-4090 Insect Rearing Cage 47.5 × 47.5 × 47.5 cm’, MegaView Science Co. Ltd., Taichung, Taiwan) at 26 °C, 70% RH, and a 16L:8D photoperiod. Nymphs and adults were provided with combinations of beans, maize, peanuts and carrots, which were replaced twice a week. A variety of branches from different tree species (e.g. *Sorbus aucuparia* L., *Cornus sanguinea* L., *Prunus avium* L.) was added as food and oviposition substrate during summer. Folded black mesh was added to each rearing cage as an additional oviposition substrate.

### Rearing of* Anastatus bifasciatus*

The original colony of *A. bifasciatus* was established in 2013 from sentinel *H.* *halys* egg masses exposed near Fully, Canton of Valais, Switzerland. Adults were maintained in cylindrical plastic containers (100 × 115 mm), with a mesh top, which were placed above Petri dishes (90 × 20 mm) filled with honey water solution. Parasitoids were fed every second day with honey water via cotton wicks that bridged the Petri dishes with the rearing containers, while undiluted honey droplets were placed on top of the mesh. The containers were maintained in an incubator set at a light/temperature cycle of L 14 h/20 °C and D 10 h/15 °C. Parasitoids were provided twice a week with *H. halys* egg masses that were either fresh (< 24 h), had been stored at 10 °C for a maximum of 3 d, or frozen for up to one year at − 80 °C. Egg masses were glued to 2 × 10 cm cardboard pieces; frozen eggs were thawed for 30 min before gluing. Parasitized egg masses were removed and placed in cylindrical plastic containers (100 × 50 mm) kept at 26 °C, 70% RH, and a 16L:8D photoperiod until adult emergence. Upon the initial establishment of the laboratory colonies, specimens of *A. bifasciatus* were taxonomically identified by Lucian Fusu (University of Iasi, Romania).

### Selection, source, and rearing of non-target test species

Non-target species were selected according to the information on *A. bifasciatus* hosts available from the literature, phylogenetic relatedness and sympatry of target and non-target species, phenology, egg size, oviposition site, rareness, and accessibility (Kuhlmann et al. 2006). In total, 28 species were selected, including 19 Lepidoptera and 9 Heteroptera (Table 1). In addition, the tropical Lepidoptera *Argema mimosae* (Boisduval) (Saturniidae) and a *Rothschildia* sp. (Saturniidae), producing particularly large eggs, were offered as hosts to investigate the effect of egg size on parasitoid offspring. Non-target Heteroptera were collected as nymphs or adults at various sites in Switzerland and Austria (Table 1), whereas most Lepidoptera species were received as eggs, caterpillars, or pupae from commercial breeders. Caterpillars and pupae were reared to the adult stage to obtain eggs for testing. Some Lepidoptera were collected as adults with a light trap placed near a forest edge on the property of CABI in Delémont, Switzerland. These adults were kept in 50 × 50 × 50 cm gauze cages for oviposition and provided with honey water and their associated host plants as oviposition stimulus if necessary. Heteroptera hosts were reared in the same conditions as described for *H. halys*; however, for some species the mixed diet described above was replaced with specific host plants required for reproduction. Non-target eggs were collected daily and weighed in groups of 10 as a measurement of their size.

**Table 1:** Non-target species test list for *Anastatus bifasciatus*

Test species	Selection criteria	Oviposition site	Origin of laboratory cultures	Stage provided/collected
Order: Heteroptera				
Family: Pentatomidae				
*Halyomorpha halys* (Stål)	Target	Underside of leaves of various host plants	Zurich and Basel, Switzerland	Adults
*Carpocoris fuscispinus* (Boheman)	Close relatedness, egg size, literature host record	Leaves and stalks of Apiaceae and Asteraceae	Delémont, Switzerland	Adults
*Dolycoris baccarum* (Linnaeus)	Habitat and host plant overlap, close relatedness	Upper side of leaves	Delémont, Switzerland	Adults
*Eurydema dominulus* (Scopoli)	Close relatedness	Leaves and stalks of Brassicaceae and Apiaceae	Delémont, Switzerland	Adults
*Graphosoma lineatum* (Linnaeus)	Close relatedness	Stalks and flowers of Apiaceae	Delémont, Switzerland	Adults
*Holcostethus strictus* (Fabricius)	Close relatedness	Herbs and trees	Vienna, Austria	Adults
*Nezara viridula* (Linnaeus)	Habitat and host plant overlap, close relatedness, literature host record	Underside of leaves of various host plants	Lugano, Switzerland	Nymphs and adults
*Palomena prasina* (Linnaeus)	Close relatedness	On host plants	Delémont, Switzerland	Nymphs and adults
*Piezodorus lituratus* (Fabricius)	Close relatedness	On stems, leaves and fruits of Fabaceae	Vienna, Austria	Adults
Family: Coreidae				
*Coreus marginatus* (Linnaeus)	Relatedness, egg size	On Rumex leaves	Delémont, Switzerland	Adults
Order: Lepidoptera				
Family: Cossidae				
*Cossus cossus* (Linnaeus)	Habitat and host plant overlap	In crevices of tree bark	Commercially obtained	Eggs
Family: Endromidae				
*Endromis versicolora* (Linnaeus)	Habitat and host plant overlap	Around thin branches	Commercially obtained	Pupae
Family: Erebidae				
*Arctia caja* (Linnaeus)	Habitat and host plant overlap	Underside of host plant leaves	Delémont, Switzerland	Adults
*Catocala dilecta* (Hübner)	Overwintering as eggs		Commercially obtained	Eggs
*Catocala electa* (Vieweg)	Threatened species in Germany, overwintering as eggs		Commercially obtained	Eggs
*Lymantria dispar* (Linnaeus)	Habitat and host plant overlap, literature host record	On trunks	Commercially obtained	Eggs
*Leucoma salicis* (Linnaeus)	Literature host record	On leaves and twigs	Commercially obtained	Eggs
Family: Sphingidae				
*Smerinthus ocellata* (Linnaeus)	Habitat and host plant overlap	Underside of leaves	Delémont, Switzerland	Adults
*Sphinx pinastri* Linnaeus	Egg size, high abundance, pollinator	On needles	Delémont, Switzerland	Adults
Lasiocampidae				
*Dendrolimus pini* (Linnaeus)	Egg size, family with many literature host records	On needles	Commercially obtained	Eggs
*Euthrix potatoria* (Linnaeus)	Egg size, family with many literature host records	On grasses	Bärschwil, Switzerland	Caterpillars
*Gastropacha quercifolia* (Linnaeus)	Habitat and host plant overlap, threatened in Germany	Underside of leaves	Commercially obtained	Eggs
*Lasiocampa quercus* (Linnaeus)	Habitat and host plant overlap	Drop eggs on ground during flight	Delémont, Switzerland	Adults
*Malacosoma neustria* (Linnaeus)	Habitat and host plant overlap, literature host records	Around twigs	Commercially obtained	Eggs
*Odonestis pruni* (Linnaeus)	Habitat and host plant overlap, endangered in Germany	On leaves	Commercially obtained	Eggs
Family: Notodontidae				
*Phalera bucephala* (Linnaeus)	Habitat and host plant overlap	Underside of leaves	Delémont, Switzerland	Adults
Family: Saturniidae				
*Samia cynthia* (Drury)	Habitat and host plant overlap	Underside of leaves	Commercially obtained	Pupae
*Saturnia pyri* (Denis and Schiffermüller)	Habitat and host plant overlap, declining populations	Underside of leaves	Commercially obtained	Pupae

### No-choice black box tests

The aim of this experiment was to determine whether eggs of non-target hosts were suitable for parasitoid development. Host acceptance (= successful oviposition, without considering the suitability for development) was not measured because *A. bifasciatus* females do not mark parasitized eggs, and thus, host acceptance cannot be determined non-destructively. Since the experiment could not be conducted in one day, in each experimental set-up, similar numbers of randomly selected naïve, 4-day-old mated *A. bifasciatus* females were tested simultaneously on eggs of the target *H. halys* (positive control) and the non-target species selected (Table 1). Each parasitoid female was individually exposed for 24 h to 10 eggs of either the target or non-target, which were glued to cardboard squares (35 × 35 mm) and placed inside small plastic Petri dishes (54 × 14 mm). Eggs used were either fresh (< 24 h) or had been stored at 10 °C for a maximum of 3 days to prevent development. Prior to the experiment newly hatched *A. bifasciatus* females had been kept together with males for 4 days at 26 °C, 70% RH, and a 16L:8D photoperiod to ensure mating. Two drops of pure honey and honey water solution were added to the corners of the cardboard to provide the parasitoids with food during the experiment. After 24 h, the wasps were removed, and exposed eggs were checked daily for hatched hosts or parasitoids, which were then counted and sexed. All tests were conducted at 26 °C, 70% RH, and a 16L:8D photoperiod.

### Comparison of size, offspring production, and longevity of* A. bifasciatus* reared from different hosts

To compare the fitness of *A. bifasciatus* reared on target and non-target hosts, the parameters adult size, 48-h offspring production, and longevity were measured. *Anastatus bifasciatus* offspring that had emerged from the target (controls) and five selected non-target species (*D.* *pini*, *E. versicolora*, *L.* *quercus*, *O.* *pruni* and *S.* *cynthia*) in the no-choice black box experiment were used. For the offspring production experiment, male and female *A. bifasciatus* were transferred to rearing containers described above immediately after emergence and maintained at 26 °C, 70% RH, and a 16L:8D photoperiod. Wasps that had emerged from different host species were kept separately. Four to 5 days after emergence, *A. bifasciatus* females (*n* = 2–16 per non-target host species) were individually placed in a small plastic Petri dish (54 × 14 mm) that contained 10 fresh (< 24 h old) *H. halys* eggs glued to cardboard squares (35 × 35 mm). After 24 h, the wasps were transferred to new Petri dishes with a fresh set of *H. halys* eggs for another 24 h. The number of eggs provided over 2 days was limited to 20, as preliminary experiments had shown that *A. bifasciatus* females could produce no more than 16 offspring within 48 h [average 7.63 ± 3.80 (SD)] (Stahl et al. unpublished data).

For the longevity experiment, pairs of male and female wasps (*n* = 5–30 per host species), each originating from the same non-target host species or *H. halys* (control), were transferred immediately after emergence to plastic tubes (7 cm height and 3.3 cm diameter) with a mesh top. Tubes were placed in an incubator set at a light/temperature cycle of L 14 h/20 °C and D 10 h/15 °C. Wasps were provided with honey water solution, and mortality of wasps was recorded daily until all wasps had died. Dead parasitoid wasps were placed in 2-ml microtubes and submerged in 96% ethanol.

Photographs were taken of randomly chosen, stored specimens with a digital microscope to compare the size of offspring from different hosts and relating size to longevity. The length of their left hind tibia was then measured using the image processing program ImageJ (Rasband 1997). For each host species, twelve male and female parasitoids were measured; if fewer *A. bifasciatus* offspring had emerged, all available wasps were used.

### Statistical analyses

The proportion of *A. bifasciatus* females producing offspring on target and non-target hosts in the no-choice black box test was calculated by dividing the number of replicates producing offspring by the total number of replicates conducted. Pairwise comparisons between each non-target species and its respective *H. halys* control were made using a generalized linear model (GLM) of the binomial family (link = logit). For some non-target species, no parasitoid females produced offspring. In these cases of complete separation of factor levels, standard methods of logistic regressions are not able to estimate coefficients. For that reason, logistic regression with Firth’s bias correction (Heinze and Schemper 2002) was used.

The relationship between the size of the parasitoid offspring and the size of the host eggs they emerged from was measured with a linear regression for females and with a logarithmic regression for males.

Sex ratio was calculated as the percentage of female *A. bifasciatus* offspring for each single egg mass, which were then averaged for each host species. It was tested for dependence on host egg size with logistic regression using GLM (family = binomial, link = logit), number of female offspring as dependent, and the average weight of host eggs as independent variable.

The fecundity of F1 generation females was determined by their offspring production during 48 h. The relationship between the host species the tested parasitoid females emerged from and their offspring production was assessed pairwise with a GLM (family = Poisson, link = log).

Longevity of female offspring of selected host species was defined as number of days until death. The influence of the host species the parasitoids emerged from on their longevity was assessed pairwise for each non-target species and their respective *H. halys* controls with a log-rank test.

All statistics were carried out with R version 3.2.3 (Team 2014) using the development environment RStudio (Team 2017). For the pairwise comparisons of the proportion of *A. bifasciatus* females producing offspring with Firth’s bias correction the package ‘logistf’ (Ploner 2010) was used and for the Tukey’s post hoc ‘multcomp’ (Hothorn et al. 2008) was applied.

## Results

### No-choice black box tests

**Fig. 1:**
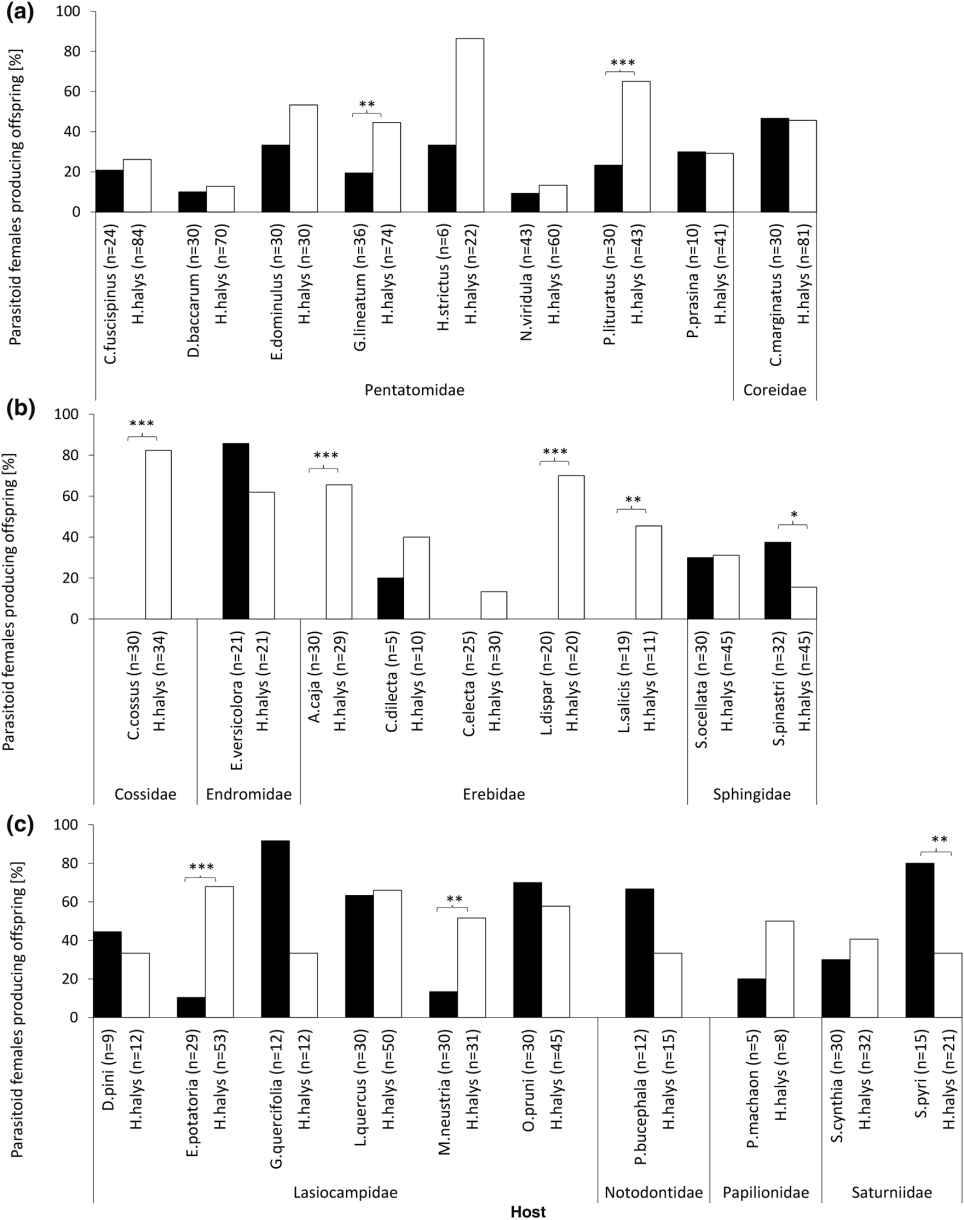
Percentage of *A. bifasciatus* females producing offspring from heteropteran (**a**) and lepidopteran (**b**, **c**) non-target hosts (black bars). White bars represent the respective *H. halys* controls. *N* states the number of tested females. Asterisks indicate significant differences between treatment and control (GLM, family = binomial)

Overall, 46.5% of *A. bifasciatus* females tested produced offspring when offered *H. halys* egg masses (controls). Even though standardized females were used, values for control replicates yielding parasitoid offspring were highly variable, ranging from 13 to 86% (Fig. 1), and dependent on the testing dates and cohorts of females used.

All ten heteropteran hosts exposed in no-choice tests produced viable *A. bifasciatus* offspring, including the target *H. halys* (Fig. 1a). Only in two species, *G. lineatum* and *P. lituratus*, the proportion of females producing viable offspring was significantly lower (binomial GLM, df = 108, *χ*^2^ = 6.707, *p* = 0.00960; df = 71, *χ*^2^ = 12.5, *p* < 0.001, respectively) than in the *H. halys* controls (Online Resource 1). Regarding lepidopteran hosts, fourteen out of nineteen tested species were suitable for parasitoid development. The only species tested from the family Cossidae (*C. cossus*), and four out of five hosts in the family Erebidae did not produce any offspring (Fig. 1b). Tested species within the families Endromidae, Sphingidae, Lasiocampidae, Notodontidae, Papilionidae, and Saturniidae were all suitable hosts, and for the majority of species the proportion of females producing offspring was as high as in *H. halys* controls (Fig. 1b, c). When offered eggs of *S. pinastri* and *S. pyri*, significantly more females produced offspring than in *H. halys* controls (binomial GLM, df = 75, *χ*^2^ = 4.70, *p* < 0.001 and df = 34, *χ*^2^ = 7.53, *p* = 0.006062, respectively), whereas for *M. neustria* and *E. potatoria* it was the opposite (binomial GLM, df = 59, *χ*^2^ = 10.18, *p* = 0.00141 and df = 80, *χ*^2^ = 26.61, *p* < 0.001, respectively) (Online Resource 1).

### Comparison of size, sex ratio, offspring production, and longevity of* A. bifasciatus* reared from different hosts

**Fig. 2:**
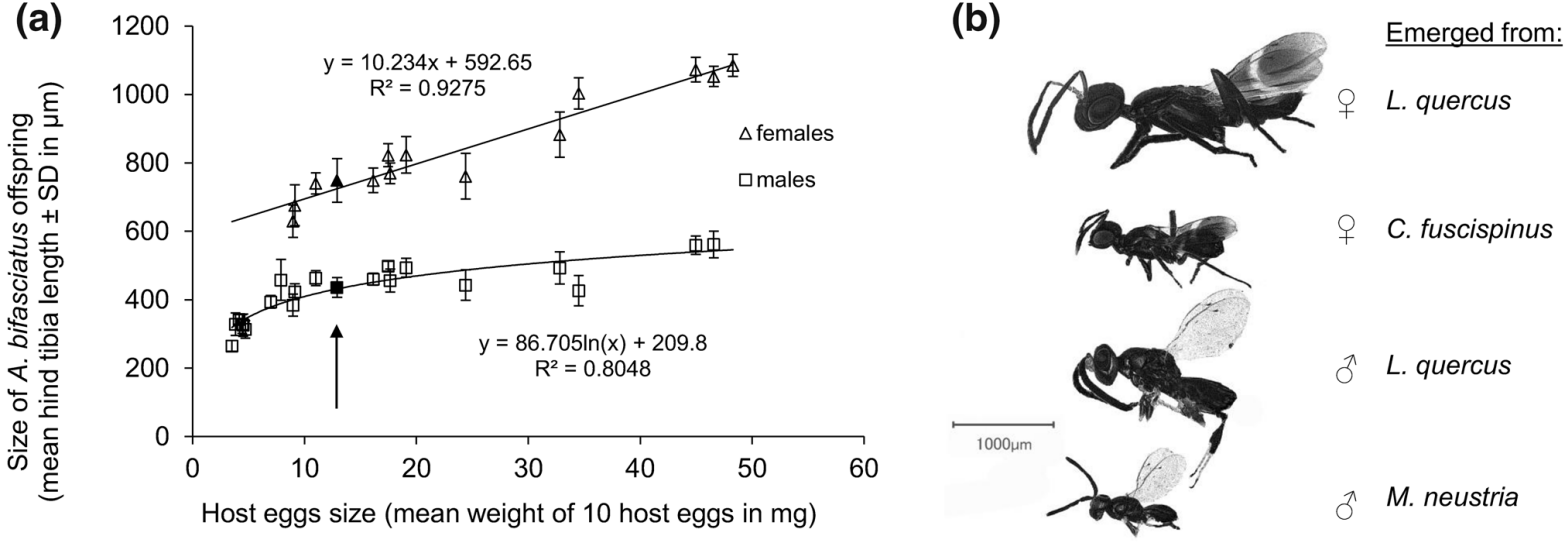
**a** Relationship between egg size of different host species, shown as mean weight of 10 host eggs, and the size of the *A.* *bifasciatus* offspring (for host species with ≥ 5 measurements), shown as mean hind tibia length. Black symbols (arrow) represent the values for the target *H.* *halys*; **b** sexual dimorphism and influence of host egg size on *A. bifasciatus* adult size (see Table 2)

A wide range of offspring sizes as well as a pronounced sexual dimorphism with larger females and smaller males was observed for *A. bifasciatus* when reared from various European hosts (Table 2 and Fig. 2b). The average male hind tibia lengths ranged from 267 ± 19 μm (host: *M.* *neustria*) to 582 ± 23 μm (host: *L.* *quercus*), while in females it ranged from 645.5 ± 46 μm (host: *C.* *fuscispinus*) to 1084 ± 28.5 μm (host: *L.* *quercus*). Even bigger females (1119.5 ± 10 μm) emerged from *A.* *mimosae*, a large tropical species. The size of the male and female offspring significantly increased with host egg size (logarithmic regression, df = 19, *t* = 8.851, *p* < 0.001, *R*^2^ = 0.80 and linear regression, df = 12, *t* = 25.505, *p* < 0.001, *R*^2^ = 0.93, respectively) (Fig. 2a). Among the heteropteran hosts tested, *H. halys* produced the largest females (Table 2), although still smaller than those emerging from most lepidopteran hosts.

**Table 2:** Mean weight of ten host eggs as estimate of host egg size, sex ratio, and average hind tibia lengths of male and female *A. bifasciatus* reared from European non-target species, two large tropical host species (*), and the target host *H. halys*

Order	Host species	# Groups of 10 eggs weighed (*n*)	Mean weight of ten eggs ± SD (mg)	Sex ratio (mean ± SD)			Average hind tibia length ± SD (µm)			
				*n*	Males	Females	*n*	Males	*n*	Females
Lepidoptera	*L. salicis*	10	2.74 ± 0.28	0			0		0	
	*M. neustria*	10	3.53 ± 0.28	8	1.00 ± 0.00	0.00 ± 0.00	11	265 ± 16	0	
	*A.caja*	10	4.48 ± 0.19	0			0		0	
	*C.electa*	10	4.47 ± 0.41	0			0		0	
	*L. dispar*	10	7.89 ± 0.43	0			0		0	
	*C. cossus*	10	8.92 ± 0.78	0			0		0	
	*O. pruni*	19	16.14 ± 1.02	89	0.73 ± 0.37	0.27 ± 0.37	12	460 ± 17	8	749 ± 36
	*E. versicolora*	10	17.47 ± 1.09	56	0.60 ± 0.38	0.40 ± 0.38	12	498 ± 15	12	823 ± 34
	*G. quercifolia*	10	17.66 ± 0.25	33	0.70 ± 0.35	0.30 ± 0.35	11	456 ± 34	12	770 ± 30
	*S. cynthia*	10	18.42 ± 1.22	38	0.79 ± 0.42	0.21 ± 0.42	12	458 ± 60	1	872 ± 00
	*S. ocellata*	10	19.09 ± 1.36	57	0.78 ± 0.22	0.22 ± 0.22	12	494 ± 27	6	824 ± 66
	*Rothschildia* sp.*	2	24.40 ± 0.42	89	0.35 ± 0.28	0.65 ± 0.28	12	443 ± 44	12	761 ± 67
	*E. potatoria*	10	24.74 ± 0.80	3	0.67 ± 0.58	0.33 ± 0.58	2	478 ± 13	0	
	*S. pinastri*	10	32.81 ± 2.12	64	0.46 ± 0.32	0.54 ± 0.32	12	493 ± 47	10	883 ± 66
	*D. pini*	9	34.50 ± 1.07	5	0.44 ± 0.43	0.56 ± 0.43	12	427 ± 44	12	1003 ± 46
	*L. quercus*	32	44.97 ± 3.87	124	0.26 ± 0.09	0.74 ± 0.09	12	559 ± 27	12	1072 ± 36
	*S. pyri*	4	46.58 ± 2.94	58	0.22 ± 0.16	0.78 ± 0.16	9	561 ± 39	12	1053 ± 30
	A. mimosae*	10	48.28 ± 2.72	46	0.24 ± 0.24	0.76 ± 0.24	3	539 ± 30	12	1085 ± 32
Heteroptera	*D. baccarum*	9	3.45 ± 1.06	3	1.00 ± 0.00	0.00 ± 0.00	12	329 ± 33	0	
	*E. dominulus*	3	4.17 ± 0.38	70	1.00 ± 0.00	0.00 ± 0.00	12	342 ± 14	0	
	*P. lituratus*	10	4.44 ± 0.42	41	1.00 ± 0.00	0.00 ± 0.00	12	329 ± 27	0	
	*H. strictus*	8	4.53 ± 0.85	8	1.00 ± 0.00	0.00 ± 0.00	8	326 ± 32	0	
	*N. viridula*	10	4.72 ± 0.18	17	1.00 ± 0.00	0.00 ± 0.00	12	313 ± 25	0	
	*G. lineatum*	10	6.98 ± 0.42	50	1.00 ± 0.00	0.00 ± 0.00	12	394 ± 18	0	
	*C. fuscispinus*	10	8.98 ± 5.28	20	0.67 ± 0.27	0.33 ± 0.27	12	384 ± 32	7	630 ± 47
	*P. prasina*	3	9.13 ± 0.81	22	0.19 ± 0.17	0.81 ± 0.17	10	424 ± 23	12	676 ± 59
	*C. marginatus*	10	11.01 ± 0.47	40	0.49 ± 0.34	0.51 ± 0.34	12	463 ± 22	12	740 ± 31
	*H. halys*	10	12.91 ± 1.25	1079	0.51 ± 0.37	0.49 ± 0.37	12	435 ± 29	12	749 ± 64

**Fig. 3:**
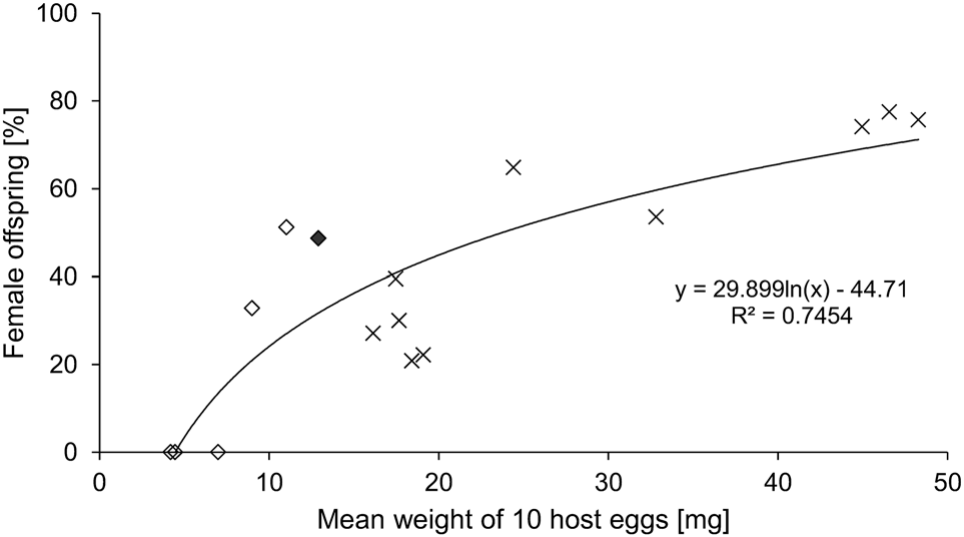
Relationship between sex ratio of *A.* *bifasciatus* offspring emerged from heteropteran (squares) and lepidopteran (crosses) non-target hosts and size of host eggs expressed as mean weight of 10 host eggs (GLM, family = binomial, *p* < 0.001). Species that generated low numbers of offspring (< 20 individuals, see Table 2) were not included in the analysis; *H. halys* is marked black

The sex ratio of the offspring was significantly affected by the size of the host (binomial GLM, df = 600, *z* = − 9.24, *p* < 0.001; Fig. 3). From eggs smaller than 0.7 mg only males emerged, while above this threshold the proportion of female offspring increased with increasing size of host eggs. The highest female-biased sex ratio (76%) was received from the large tropical lepidopteran host *A. mimosa*. The proportion of males (51%) and females (49%) emerging from *H. halys* eggs was nearly balanced.

**Fig. 4:**
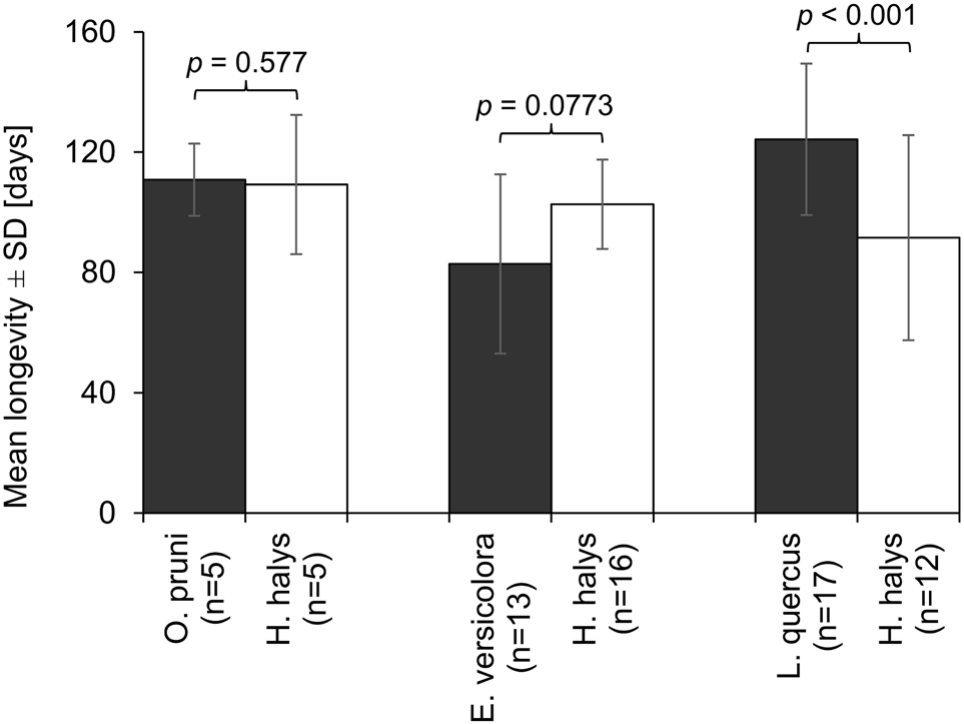
Mean longevity of *A. bifasciatus* females reared from three non-target species (black bars) and the target *H. halys* (white bars) at a light/temperature cycle of L 14 h/20 °C and D 10 h/15 °C. *N* is the number of females tested; the mean weight of ten eggs in mg (*H. halys* = 12.91, *O. pruni* = 16.14, *E. versicolora* = 17.47, *L. quercus *= 44.97) is mentioned as indicator for host egg size (see Table 2). *P* values are indicated on top of the compared pairs (log-rank test)

The longevity of individual *A. bifasciatus* females emerged from the non-target hosts *L. quercus*, *O. pruni*, and *E. versicolora* as well as the target host ranged from 7 to 172 days at a light/temperature cycle of L 14 h/20 °C and D 10 h/15 °C (Fig. 4). Those females that had emerged from the host *L. quercus*, producing the largest eggs of the species tested and accordingly the largest females, lived significantly longer than those emerged from the *H. halys* controls (log-rank test, df = 1, *χ*^2^ = 16.4, *p* < 0.001). In contrast, females emerged from *O. pruni* and *E. versicolora* did not live significantly longer than their controls (log-rank test, df = 1, *χ*^2^ = 0.3, *p* = 0.577 and *χ*^2^ = 3.1, *p* = 0.0773, respectively) (Fig. 4).

**Fig. 5:**
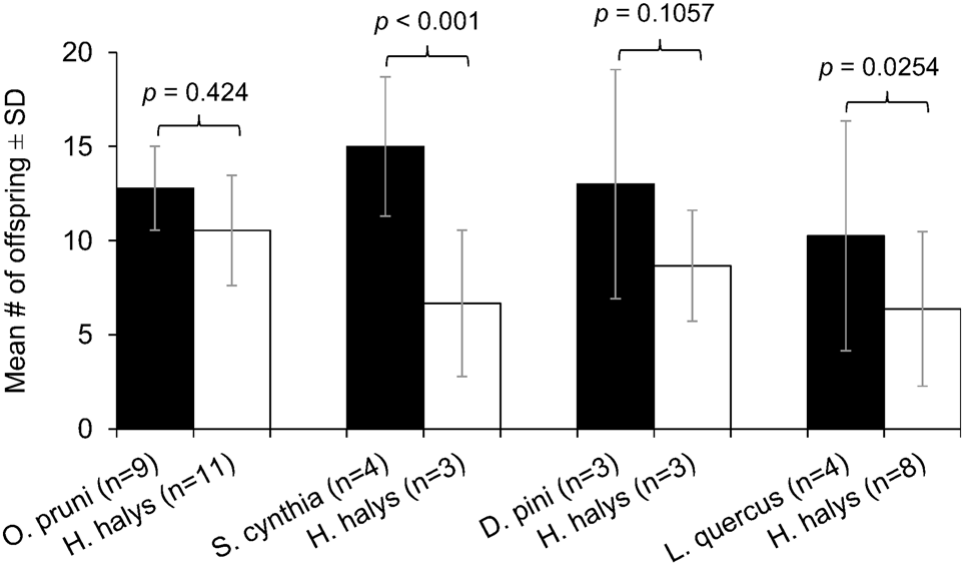
Mean offspring production of *A.* *bifasciatus* females originating from different host species, when offering 2 × 10 *H.* *halys* eggs over 2 × 24 h consecutively. Black bars represent offspring production of females emerged from non-target hosts and white bars offspring emergence of females emerged from *H.* *halys* (control). Females emerged from *H.* *halys* are smallest in size within this group (see Table 2). N states the number of tested females. *P* values are indicated on top of the compared pairs (GLM, family = gamma; post hoc Tukey)

The offspring production of *A. bifasciatus* females emerged from non-target species, all of which produced larger females than *H. halys*, was generally higher than the offspring production of females emerged from *H. halys* (Fig. 5), but only for those emerged from *S. cynthia* and *L. quercus*, the difference was significant (Poisson GLM, df = 5, *z* = 3.14, *p* < 0.001 and df = 10, *z* = 2.26, *p* = 0.0254, respectively). For the other two non-targets, *O. pruni* and *D. pini*, the difference showed no statistical significance (Poisson GLM, df = 17, *z* = 0.799, *p* = 0.424 and df = 4, *z* = − 1.601, *p* = 0.1057, respectively). No significantly different offspring production could be found between females emerged from non-target hosts (Poisson GLM, df = 18, *z* = 1.15, *p *> 0.05).

## Discussion

Assessing the host range of potential biological control agents is a major step in risk analysis when exotic natural enemies are concerned, but also needs to be considered in inundative biological control with native candidates (van Lenteren and Loomans 2006). If large quantities of a biological control agent are released, these may affect not only the target but also populations of non-target hosts in the target habitat and surrounding habitats due to dispersal behaviour (van Lenteren and Loomans 2006).

Surveys for native natural enemies attacking the invasive brown marmorated stink bug in Europe showed that the egg parasitoid *A. bifasciatus* is a potential candidate for inundative releases to mitigate damage in European fruit orchards due to oviposition peaks of *H. halys* (Haye et al. 2014a, b). However, even though knowledge on its specific host range and host specificity is scarce, *A. bifasciatus* has been reared from more than 30 hosts in the orders Heteroptera and Lepidoptera (Noyes 2014) and thus can be considered a polyphagous parasitoid.

In laboratory no-choice tests, developmental suitability of non-target host species for *A. bifasciatus* was demonstrated by the successful production of progeny on 23 out of 28 tested non-target host species. Five out of seven species that were previously recorded as host of *A. bifasciatus* in the literature were confirmed: *N. viridula* (Genduso 1977; Jones 1988), *D. pini*, *G. quercifolia*, *M. neustria* and *S. pyri* (Herting and Simmonds 1976) (Fig. 1). In contrast, *L. dispar* and *L. salicis* (Erebidae) were not suitable hosts (Fig. 1), although they had been recorded as hosts for *A. bifasciatus* in Turkey (e.g. Avci 2009; Herting and Simmonds 1976) and in Eastern Europe (Boucek 1977; Zaharieva-Pentcheva and Georgiev 1997), respectively. In agreement with the present study, Hayat (1975) suggested that *L. dispar* is not a suitable host for *A. bifasciatus*, referring to the common confusion of *Anastatus japonicus* Ashmead with *A. bifasciatus*. Meanwhile, eight new heteropteran hosts in two families and eleven lepidopteran hosts in six families can be added to the fundamental host range of *A. bifasciatus* (Fig. 1), confirming its highly polyphagous nature.

Apart from the Cossidae family, represented by only one species, all families tested included suitable host species. Overall, the majority of non-target species tested was as suitable as the target *H. halys*, while in two cases, *S. pinastri* and *S. pyri*, the proportion of females producing offspring was even higher than for the target. Interestingly, only one out of five species within the family Erebidae produced parasitoid offspring. Similar results were found for the generalist *Trichogramma brassicae* Bezdenko (Babendreier et al. 2003a), indicating that the taxonomy of potential host species is not the only criteria of host selection for generalist parasitoids. There are various reasons why certain species may not have been suitable for parasitoid development: eggs within the families Cossidae and Erebidae were all relatively small (< 1 mg) and may not provide enough resources for successful offspring development. On the other hand, heteropteran hosts with eggs smaller than 1 mg were frequently accepted, even if only male offspring was produced. Another explanation could be that some eggs may have been protected by morphological or chemical defences as demonstrated for the moth *Zygaena trifolii* (Esper) (Lepidoptera: Zygaenidae) whose eggs contain toxic substances (Marsh and Rothschild 1974). Eggs of some Erebidae are naturally covered in either hairs (*L. dispar*), a paper like substance (*L. salicis*) or contain high concentrations of choline esters (*A. caja*), which can prevent parasitism (Morley and Schachter 1963, Ohta et al. 1994). The materials covering the host eggs of *L. dispar* and *L. salicia* were removed before the experiments, which could negate their parasitism-inhibiting effects (Darling and Johnson 1982), but residual traces may still have led to the rejection of the eggs by *A. bifasciatus.* In contrast, the only species in the family Erebidae that produced offspring was *C. dilecta,* which is not protected by any of the factors described above.

Considering the continuous rearing of the *A. bifasciatus* colony on *H. halys* for about 35 generations, a shift in host preference may have led to an underestimation of *A. bifasciatus* females producing offspring on non-target species in comparison with natural *A. bifasciatus* populations or parasitoids reared on factitious hosts. In general, the proportion of *A. bifasciatus* females producing offspring when given eggs of the target *H. halys* was extremely variable. Since only little is known on the reproductive biology of this species, it is possible that, for example, using older or experienced females may have resulted in more consistent data across the controls.

The sex ratio of *A. bifasciatus* offspring was strongly dependent on the host egg size. Lepidopteran and heteropteran hosts with an egg size below 0.7 mg only produced male offspring. In hosts with eggs ranging from 0.9 to 1.9 mg the sex ratio was heavily male biased, whereas eggs larger than 4 mg produced significantly more females. Our results agree with the theory of conditional sex allocation, where the parasitoid offspring shifts towards a female bias with increasing host quality (Charnov 1979; Charnov et al. 1981). In line with our findings, also Hou et al. (2009) reported a more female-biased sex ratio when *Anastatus* sp. was reared on eggs of oak silkworm (*Antheraea pernyi* (Guérin-Méneville), Lepidoptera: Saturniidae) instead of *H. halys*. While the sex ratio of the parasitoid offspring has no immediate effect on the efficacy of inundative releases against *H. halys*, the attack of non-target species yielding only male offspring would prevent the development of successive generations on a given non-target species, thereby limiting population-level non-target effects beyond a single generation.

For *H. halys*, the heteropteran species with the largest eggs tested (1.29 mg), the sex ratio was balanced, indicating that *H. halys* is a medium-quality host. However, most similar sized lepidopteran eggs yielded a less female-biased sex ratio of about 20–40% female offspring, providing some evidence that the quality of lepidopteran eggs is assessed as less good by *A. bifasciatus* compared to heteropteran hosts. Obviously, host quality was strongly correlated with host size, since larger host eggs yielded larger parasitoid offspring, which in turn profit from fitness advantages over smaller conspecifics (Arakawa et al. 2004; Medal and Smith 2015). The sexual dimorphism of *A.* *bifasciatus* with larger females and smaller males was more pronounced with increasing host egg size. A stronger correlation was observed for females, which may be explained by the fact that female fecundity is more dependent on size than male mating success (Morris and Fellowes 2002; Van den Assem 1970).

In many host–parasitoid systems female fecundity and longevity are positively correlated with female body size (Godfray 1994). In addition, Visser (1994) showed a positive correlation between female size and other fitness parameters like egg-carrying capacity, egg size, and females’ searching efficiency for the parasitoid *Aphaereta minuta* (Nees) (Hymenoptera: Braconidae). All of these are desirable attributes for biological control agents, and accordingly releasing ‘fitter’ females may increase the success of inundative biological control. In the present study, we did not measure the lifetime fecundity of different sized females, but it was demonstrated that larger females, which had emerged from larger lepidopteran eggs, tended to produce more offspring within 48 h than their smaller conspecifics emerged from *H. halys* eggs. In addition, females reared on larger lepidopteran hosts (shown for *L. quercus*) lived longer than smaller females reared from *H. halys* eggs. Consequently, using lepidopteran hosts with eggs much larger than those of *H. halys* for mass producing *A. bifasciatus* may be the better approach to maximize the fitness of the solitary parasitoids and thus the effectiveness of inundative releases, an approach that was also followed for rearing other *Anastatus* spp. against heteropteran pests (Fay and De Faveri 1997; Huang et al. 1974). However, rearing a biological control agent on an alternative host may also alter its host preference (Corrigan and Laing 1994; Henter et al. 1996).

Past examples suggest that even polyphagous parasitoids, which showed a broad host range in the laboratory, can be used for inundative releases because the parasitoid’s behaviour in the field indicates low risks for non-target species (Babendreier et al. 2003a, b; Haye et al. 2005). Even if non-target species are attacked not only in laboratory conditions but also in the field, it does not necessarily translate to a threat to their population (Van Lenteren et al. 2006; van Lenteren and Loomans 2006). A key feature in the assessment of risks in the field under these circumstances is dispersal behaviour, which still is not well understood for *A. bifasciatus*. In case future studies show that it will disperse frequently from the release area into natural adjacent habitats, negative impacts on some non-target species seem possible. Our data suggest that particularly large lepidopteran hosts such as *L. quercus* and the endangered *S. pyri*, producing a heavily female-biased offspring, would be ‘optimal’ hosts and could be negatively affected. In contrast, species producing eggs with a weight of 0.7 mg or smaller, including nearly all heteropteran non-target species tested, would only produce male offspring and would not contribute to population growth of the parasitoid. Even though an exact threshold is difficult to calculate, our data suggest that any non-target species having eggs smaller than 0.3 mg would not be accepted as host and thus not at risk.

Besides these direct effects, the arrival of an invasive species such as *H. halys* may also indirectly affect populations and communities of native species via the sharing of natural enemies, a process referred to as apparent competition (Holt 1977). Since *A. bifasciatus* is native to Europe and regularly reared from *H. halys* in the field, it is possible that in regions where *H. halys* densities are particularly high, the parasitoid will build up large populations on the exotic hosts, which then may also cause declines of local insect communities. Although apparent competition is recognized as an important factor structuring insect communities (e.g. Bompard et al. 2013; Bonsall and Hassell 1998; Morris et al. 2004), increasing *A. bifasciatus* populations have not yet been observed in areas invaded by *H. halys*. Continuous inundative releases may accelerate local population growth, making apparent competition more likely, particularly if highly suitable lepidopteran hosts are present in the area of release or nearby.

A better understanding of the predictability of such ecological interactions between *H. halys* and *A. bifasciatus* can only be accomplished by increasing our knowledge on the reproductive biology, behavioural ecology, and dispersal of *A. bifasciatus*. The results of this study indicate that non-target effects cannot be ruled out, but expanding the laboratory non-target studies to semi-field and field conditions will help to better understand potential risks of inundative releases of this native parasitoid.

## Author contribution

DB, JS, and TH conceived and designed research. JS and TH conducted experiments. JS analysed data. JS wrote the manuscript, and DB and TH edited it. All authors read and approved the manuscript.

## Electronic supplementary material

Below is the link to the electronic supplementary material.

Supplementary material 1 (PDF 137 kb)
