# Supplementary material for: Using the egg parasitoid Anastatus bifasciatus against the invasive brown marmorated stink bug in Europe: can non-target effects be ruled out?
Source: J Pest Sci (2004). 2018 Mar 21;91(3):1005–17. doi: 10.1007/s10340-018-0969-x (PMC5978841; doi:10.1007/s10340-018-0969-x)
Supplement: Supplementary file 1 — Supplementary material 1 (PDF 137 kb) [file 10340_2018_969_MOESM1_ESM.pdf]

Title: **Using the egg parasitoid *Anastatus bifasciatus* against the invasive brown marmorated stink bug in Europe – can non-target effects be ruled out?**

Journal of Pest Science

Authors: Judith Stahl[1,2]*, Dirk Babendreier[1], Tim Haye[1]

[1] CABI, Rue des Grillons 1, 2800 Delémont, Switzerland
[2] Institute of Ecology and Evolutionary Biology, University of Bremen, Leobener Str. NW2, 28359 Bremen, Germany
* Email: j.stahl@cabi.org

Electronic Complementary Data: Statistical results from bias-corrected logistic regression testing differences between the proportions of females producing offspring when offered either a non-target species or *H. halys* (control)

Corresponding Figure	Non-target species	Estimate	SE[a]	χ^2 statistic	*P* value
1a	*C. fuscispinus*	-0.2440	0.551	0.20817	0.6482
	D. baccarum	-0.1937	0.675	0.08644	0.7688
	E. dominulus	-0.8056	0.525	2.44818	0.1152
	G. lineatum	-1.1553	0.476	6.70782	0.00960
	H. strictus	-2.3054	1.038	6.05309	0.01388
	N. viridula	-0.3515	0.626	0.32955	0.56592
	P. lituratus	-1.7512	0.533	12.4492	< 0.001
	P. prasina	0.0965	0.760	0.01736	0.89517
	C. marginatum	0.0419	0.429	0.00984	0.9210
1b	*C. cossus*	-5.0982	1.500	42.4568	< 0.001
	E. versicolora	1.2024	0.746	2.93270	0.0868
	A. caja	-4.1776	1.496	23.5297	< 0.001
	C. dilecta	0.0770	1.178	0.00500	0.9436
	C. electa	-1.0201	1.002	1.20904	0.2715
	L. dispar	-4.5159	1.544	24.0357	< 0.001
	L. salicis	-3.4965	1.589	9.65659	0.00189
	S. ocellata	-0.1115	0.491	0.05296	0.81800
	S. pinastri	1.1410	0.544	4.69441	0.03026
1c	*D. pini*	0.4353	0.904	0.25544	0.6132
	E. potatoria	-2.7659	0.649	26.6149	< 0.001
	G. quercifolia	2.6728	1.088	8.57731	0.00340
	L. quercus	-0.2095	0.484	0.19204	0.66122
	M. neustria	1.8356	0.631	10.1888	0.00141
	O. pruni	0.5100	0.498	1.10102	0.29404
	P. bucephala	1.2826	0.815	2.80546	0.09394
	P. machaon	-1.0986	1.252	0.96568	0.32576
	S. cynthia	-0.4490	0.535	0.73429	0.39150
	S. pyri	1.9322	0.776	7.53158	0.00606

[a] standard error of the coefficient estimate
